# Characterization of the 18S rRNA Gene for Designing Universal Eukaryote Specific Primers

**DOI:** 10.1371/journal.pone.0087624

**Published:** 2014-02-07

**Authors:** Kenan Hadziavdic, Katrine Lekang, Anders Lanzen, Inge Jonassen, Eric M. Thompson, Christofer Troedsson

**Affiliations:** 1 Department of Biology, University of Bergen, Bergen, Norway; 2 Uni Computing, Uni Research AS, Bergen, Norway; 3 Department of Ecology and Natural Resources, NEIKER-Tecnalia, Derio, Spain; 4 Department of Informatics, University of Bergen, Bergen, Norway; 5 Sars International Centre for Marine Molecular Biology, University of Bergen, Bergen, Norway; 6 Uni Environment, Uni Research AS, Bergen, Norway; King Abdullah University of Science and Technology, Saudi Arabia

## Abstract

High throughput sequencing technology has great promise for biodiversity studies. However, an underlying assumption is that the primers used in these studies are universal for the prokaryotic or eukaryotic groups of interest. Full primer universality is difficult or impossible to achieve and studies using different primer sets make biodiversity comparisons problematic. The aim of this study was to design and optimize universal eukaryotic primers that could be used as a standard in future biodiversity studies. Using the alignment of all eukaryotic sequences from the publicly available SILVA database, we generated a full characterization of variable versus conserved regions in the 18S rRNA gene. All variable regions within this gene were analyzed and our results suggested that the V2, V4 and V9 regions were best suited for biodiversity assessments. Previously published universal eukaryotic primers as well as a number of self-designed primers were mapped to the alignment. Primer selection will depend on sequencing technology used, and this study focused on the 454 pyrosequencing GS FLX Titanium platform. The results generated a primer pair yielding theoretical matches to 80% of the eukaryotic and 0% of the prokaryotic sequences in the SILVA database. An empirical test of marine sediments using the AmpliconNoise pipeline for analysis of the high throughput sequencing data yielded amplification of sequences for 71% of all eukaryotic phyla with no isolation of prokaryotic sequences. To our knowledge this is the first characterization of the complete 18S rRNA gene using all eukaryotes present in the SILVA database, providing a robust test for universal eukaryotic primers. Since both *in silico* and empirical tests using high throughput sequencing retained high inclusion of eukaryotic phyla and exclusion of prokaryotes, we conclude that these primers are well suited for assessing eukaryote diversity, and can be used as a standard in biodiversity studies.

## Introduction

Next generation sequencing technology has made high-resolution biodiversity assessments of environmental samples possible, generating a large number of sequence reads available for analysis after a single run [1]. In particular, the 454 pyrosequencing technology has been preferred in recent biodiversity studies [2]–[5], partly because bioinformatic tools for analyzing the data have become more accessible and easier to use [6]–[8]. Most biodiversity studies have targeted portions of phylogenetically useful genes (taxonomic “markers”), limited to a few hundred nucleotides. This is because the technology does not readily allow full gene characterization due to sequence length restrictions. In order to obtain an accurate estimate of the biodiversity from a sample it is therefore necessary to attain sequences from a sufficiently variable region of the target phylogenetic gene. One common marker is the small subunit of the ribosomal RNA gene (SSU rRNA), whose sequence and structure has been characterized and contains nine highly variable regions; V1 to V9 [9]–[12]. Although the SSU rRNA is present in all living cells with a highly conserved function, there are some distinct differences between its sequences in eukaryotes compared to prokaryotes. While the V6 region has been considered variable and well-suited for prokaryotic studies of biodiversity [13], [14], this region is more conserved in eukaryotes and therefore often avoided [10]. The V4 region on the other hand is the largest variable region in eukaryotes [15], while being shorter in prokaryotes. Nevertheless, several studies have applied this region in assessments of the composition of microbial communities [16], [17]. For studies of eukaryotic diversity, several variable regions have been suggested, with the V4 and V9 being the most prominent candidates [3]–[5], [18]. However, the successful application of a variable region to a biodiversity study also depends on the amplicon length as well as viable primer sites flanking the variable region [19].

The choice of primers will impact results from biodiversity assessment of an ecosystem [20] and there are some important considerations in primer design. The universality of primers will determine the upper limit of inclusion in a biodiversity assessment, but complete universality introduces loss of resolution. Using primers that target all prokaryotes and eukaryotes limits the depth of biodiversity assessment of both groups. Limiting the universality of the primers might, on the other hand, exclude important groups in the analysis, and introduce biases, favoring some organisms or groups [21], [22]. Furthermore, the use of different universal primers makes direct comparison between studies more challenging. This places important constraints on the interpretation of results for purposes such as environmental monitoring. One possible way to address this challenge is the use of phylogenetic placement [23], [24]. By using whole sequences from the 18S rRNA gene as a reference tree one can compare sequences originating from different regions. However, the bias from the lack of complete sequences in the reference tree will still affect the results. A large number of universal eukaryotic primers targeting different region of the 18S rRNA gene have been used previously (Table S2), but their universality has not been properly assessed. A recent study tested universality of published prokaryote specific primers [25] and several subgroups of eukaryotes have received attention regarding the use of group-targeted primers in the assessment of biodiversity [26]–[29]. In addition, the choice of variable region of the SSU rRNA gene for eukaryotic diversity estimates is highly related to finding the best set of primers which can also provide a better level of standardization in future studies.

In this study we have performed a complete characterization of the variable and conserved regions within the 18S rRNA gene using the publicly available SILVA database containing more than 500,000 non-redundant SSU rRNA sequences. We mapped available universal primers from the literature as well as self-designed primers, and evaluated them based on their suitability for eukaryotic biodiversity studies. This generated 100 non-degenerate eukaryote primers distributed along the entire 18S rRNA gene sequence. When choosing the optimal primer pair, a predetermined set of testing parameters adapted to environmental monitoring studies with unknown organisms was used. The results from these studies produced a primer pair well suited for eukaryotic biodiversity studies, which was tested empirically using a high throughput sequencing approach on a diverse set of marine sediment samples.

## Methods

### Primer Design and 18S rRNA Gene Characterization

Our primary goal when designing universal eukaryotic primers was to cover the broadest possible spectrum of eukaryotes while completely excluding prokaryotes. To facilitate the choice of primers, we used the full SILVA database v.106 [30], [31] containing a total of 555,530 sequences with 499,367 prokaryotic and 56,163 eukaryotic sequences. These were all pre-aligned in the SILVA database, and the alignment formed the basis for the full characterization of the 18S rRNA gene. In the characterization of the 18S rRNA gene, all prokaryotic sequences from the database were removed. The distinction between prokaryotes and eukaryotes was based on the SILVA alignment and annotation, as well as taxonomic annotation from the National Center for Biotechnology Information (NCBI) database also included in SILVA (v.106). Although the taxonomic annotation of sequences based on SILVA alignment and NCBI mostly coincided, there were some notable exceptions. In the SILVA database 55,937 sequences were annotated as eukaryotes, while the remaining 226 sequences had an ambiguous classification, which suggested that the sequences could originate from either prokaryotes or eukaryotes. In those cases the annotation resulting from the alignment was given preference. After the removal of prokaryote sequences, many columns contained only gaps in the SILVA alignment. These columns contained no relevant information for the characterization of the 18S rRNA gene and were subsequently removed. The resulting alignment of eukaryotes contained 24,793 positions, and was mapped to the 1,800 base pair (bp) long 18S rRNA gene of the yeast, *Saccharomyces cerevisiae* (accession number Z75578). The exclusion of all gaps in the reference sequence generated 13-fold reduction in length of the alignment. Since gap removal could potentially remove some information, the relevance of the estimated information was estimated by measuring the percentage of nucleotides present in the gap positions. The information loss was minor since it predominantly consisted of gaps. The estimation of the amount of removed information is presented in Figure S1. For each position in the final alignment, a Shannon entropy value was calculated [32] based on the abundance of the four nucleotides according to:
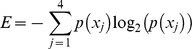
where *p*(*x_j_*) was the frequency of the nucleotide *x_j_* in the alignment (*j*). Ambiguous nucleotides in the database were also taken into account [33]. The resulting entropy values were plotted along the length of the *S. cerevisiae* 18S rRNA gene, where high values indicated variable regions and low values indicated conserved areas. Regions of some minimum length with low values constitute possible target sites for designing new universal primers. In order to reduce the search space and hence computation time, potential target sites were defined as a block of ≥10 alignment positions with ≥90% of the positions having a Shannon entropy values under 0.2. Once these regions were defined, potential universal eukaryote primers were identified by extending the sequence in both directions. Each nucleotide extension was tested against the SILVA database. Since each extension also decreased coverage within eukaryotes, the results from each extension were evaluated as a compromise between maximal coverage of eukaryotes and minimal inclusion of prokaryotes. In addition to the extension process, the constraints on primer length (18–22 nucleotides) were respected. In the final selection, the importance of the 3′ end was addressed by considering its nucleotide content. Primers having two or three G or C out of the five nucleotides at the 3′end were given preference. All modifications to the primers were followed by a new search against the full SILVA database. The search for reverse primer candidates was done using the same method but applying it to complementary sequences of the potential target sites. Universal eukaryotic primers from the literature [34]–[50] were also treated as potential target sites and, after reducing degeneracy when needed, processed by the same pipeline.

When testing and evaluating each primer, the following parameters were considered:

1. Primers with exact matches to prokaryotic sequences in the SILVA database were excluded. However, primers matching a large number of eukaryote sequences and low number of prokaryotes were kept for further testing.
2. Preference was given to primers with a high number of matches to eukaryotes in the database. Furthermore, representation of different rank groups i.e. genera or phyla were tested. Ideally, all eukaryote phyla should be represented.
3. Degeneracy of the primers was avoided allowing maximal stringency in the PCR optimization to reduce discrepancy between *in silico* and *in situ* analyses and avoid biased template-to-product ratios in the amplification.
4. Primer lengths between 18–22 nucleotides (nt) were selected. When departing from this constraint, primers shorter than 18 nt that effectively excluded non-target groups (prokaryotes) were preferred compared to longer primers above 22 nt, since longer primers will decrease eukaryotic coverage.
5. When pairing the forward and reverse primers, the average length of the generated amplicons was optimized to fit criteria for current 454 pyrosequencing technology (i.e. optimally 500–650 bp, but always <800 bp including primers, tags and adapters)
6. For high stringency hybridization of the primers to the template sequence in the PCR assay, we aimed at having at least two of the last three nucleotides in the 3′-end of the primer being guanine or cytosine as these nucleotides have a stronger binding energy than adenine and thymine.
7. To avoid non-optimal PCR assays, the difference in T_m_ between the two primers (**Δ**T_m_) was kept below 5°C.
8. The candidate primers were tested *in silico* in order to avoid hairpins and self-dimerization.
9. Priorities were given to primers amplifying the 18S rRNA gene variable regions with high taxonomic information.

An important factor worth considering when designing primers is possible mismatches between the primer and its template. A sequence mismatch will likely affect the results of the PCR amplification, through the efficiency and stability of binding between primers and template, potentially leading to amplification failure. Mismatches in the primer will therefore be useful to exclude undesired groups. However, a single mismatch may not be sufficient to exclude groups. Consequently, it is desirable to have non-targeted groups excluded with at least two mismatches in the primer design, and to find highly conserved DNA segments specific to the target group. When searching for the most adequate primer pair, the robustness of the primers in prokaryote exclusion was also tested allowing both one and two mismatches.

### Biodiversity Verification

The optimal universal eukaryotic primers selected as described above were tested *in silico* using the SILVA database and empirically by high throughput sequencing (454 pyrosequencing). Conserved nucleotide segments in most eukaryotes were the primary target when developing the primers and consequently the total number of generated hits was evaluated. However, even though the database used here contained sequences originating from a large and diverse set of projects targeting a wide variety of eukaryotic groups, it still only contains a small fraction of the true biodiversity expected in nature. In addition, the coverage of different groups across eukaryotic taxonomy is unevenly distributed. Our *in silico* data on primer inclusion indicated a 84%, 85%, 90%, 95% and 87% coverage at the genus, family, order, class and phylum levels, respectively, in the SILVA database. Because of the potential bias of the database we have evaluated the test of coverage at the phyla level as most reliable, since this level of phylogenetic resolution will be less sensitive to uneven entries of sequences. Nevertheless, the bias of the database regarding the unevenly distributed coverage of phyla is still present. The sequences were clustered using an identity scale from 100% to 95%, and the taxonomy from NCBI was used to evaluate the adequacy of amplicons for representing biodiversity. A similar type of analysis was further applied using all the highly variable regions of the 18S rRNA gene. This generated data on the applicability of each highly variable region for biodiversity studies, independent of primer sites found according to the criteria described above.

To evaluate whether the chosen primers would yield an adequate representation of biodiversity on environmental samples, an empirical test using samples of three different marine sediments from a Norwegian offshore environmental monitoring program in the North Sea (Region III, 2010) was performed. The samples originated from the oil fields Oseberg C, Huldra and Fram Vest on the Norwegian continental shelf and were of different sediment composition; fine sand, coarse sand and clay, respectively. The sediment samples in this study were provided by “Det Norske Veritas (DNV)” with permission from Statoil. No specific regulatory permission was required for these samples as they were part of an already ongoing monitoring program. The samples did not involve endangered or protected species. Assuming that the different geological characteristics of the sediments will host different fauna, thereby giving a broader test of eukaryote coverage, the primers were empirically tested on extracted DNA from all three sediments, and the data were analyzed from the combined, pooled, sequences. For each sampling station, approximately 50–100 g of sediment was transferred to a 250 ml plastic container (Kautex Textron) and fixed with 96% ethanol. This resulted in a final concentration of approximately 80% ethanol due to water content of the sediment. The samples were stored at −20°C until further analysis. From each sediment, genomic DNA was extracted from ten replicates of 0.5 g sediment (5 g in total) using MoBio power soil DNA extraction kit (Mo-Bio Laboratories Inc.), which had been found to be optimal for different marine sediments (unpublished data). The PCR amplification was performed using 25 µl Hot Start Taq Master Mix (Qiagen), 0.5 µM of each universal primer (F-566 and R-1200) with a sequencing tag and an adaptor for 454 pyrosequencing attached, and 1 µg/µl of BSA to reduce the effect of inhibitors from the sediments. PCR amplification was carried out in a thermal cycler (C1000™ Thermal Cycler, BioRad) using the following program; 95°C for 15 min, 35 cycles consisting of 95°C for 45 sec, 60°C for 45 sec, 72°C for 1 min, and a final extension step of 72°C for 10 min. The amplicons were visualized by a 1.5% agarose gel electrophoresis stained with gelred (Biotium). GeneRulerT DNA Ladder Mix (Fermentas) was added to the gel as a size marker. To retrieve enough material for the sequencing reaction and to avoid technical artifacts, eight PCR reactions were run per sample. After amplification, PCR replicates were pooled together and concentrated by using a vacuum centrifuge. Concentrated DNA was purified to remove the primers and other compounds using Agencourt AMPure XP (Beckman Coulter Inc.) following manufacturers recommendations. DNA concentrations were determined by QuantiT™ PicoGreen® dsDNA quantitation kit (Invitrogen) together with a Nanodrop Fluorospectrophotometer (ND 3000 Spectrophotometer, Nanodrop Technologies Inc.). Bacteriophage λ DNA (Invitrogen) was used to produce the DNA standard curve. Pyrosequencing was performed on a GS FLX instrument using Titanium chemistry at GATC Biotech (Konstanz, Germany). Samples from the three sediments were sequenced on a single picotiter plate (PTP) divided in separate sections, one for each sediment. The sequencing produced 860,390 reads. After filtering low quality reads there were 508,712 reads left having an average length of 383 nt with minimum and maximum length of 201 and 579 nt respectively. Data from the 454 pyrosequencing of the marine sediments are available at the NCBI Sequence Read archive, with accession number SRP033298.

### Software

The SILVA database v.106 and ARB software v. 5.2 were used to identify and test specificity of the different primers *in silico* [30], [31], [51]. An internally developed AmpliconGenerator v.0.1 (http://sf.net/projects/amplicongenerator) was used to characterize nucleotide variability along the reference sequence. The software was also used to generate *in silico* amplicons and to assess the conservation of biodiversity as a function of sequence similarity. IDT SciTools OligoAnalyzer 3.0 (Integrated DNA Technologies, Coralville, IA) was used to estimate melting temperatures of the primers as well as the presence of hairpins, self- and hetero-dimers in the candidate primers. Sequences from the environmental samples were processed through the bioinformatic pipeline AmpliconNoise according to Quince and co-workers [6]. As a first step in the pipeline, low quality reads were removed, defined as shorter than 200 nt or having inadequate signal intensity. Subsequently, noise from the flowgram and PCR-generated errors were removed using established probabilistic iterative algorithms [6], [52] and chimeras removed using the program Perseus [6]. The reads were then clustered together using the complete linkage-clustering algorithm implemented in FCluster, based on pairwise distances as calculated by NDist [6], and aligned to the SILVA and NCBI databases using BLAST for the purpose of taxonomic annotation. The same software package was also applied to build operational taxonomic units (OTUs). Since the choice of the pipeline can influence the results of metagenetic studies and because there is no consensus on which pipeline to use, the results produced with AmpliconNoise were compared to results produced by an alternative commonly used pipeline, QIIME. In QIIME, original reads were filtered based on the quality score, but no further denoising procedure was applied. Further, in order to remove potential chimeras, OTUs representing singletons were discarded.

### Taxonomic Annotation

The SILVA database (v.106) taxonomy was used as reference when testing the primers. The database provides two taxonomic identitifiers for each sequence, one uses maximum parsimony method to assign taxonomy to an aligned sequence and the other states the NCBI assigned taxonomy. However, for the purpose of designing universal eukaryotic primers the difference between the two taxonomies in the database version used was marginal, only 226 out of 55937 had inconsistent taxonomy at kingdom level. In those cases the taxonomy based on the sequence alignment in the database was the prefered choice. In the evaluation of primer coverage within eukaryotes, phyla from NCBI were used as a grouping level. A phylum was considered covered if the primer pair had a perfect match to a sequence belonging to the group. Since eukaryote phyla are unevenly represented in the database, ranging from a few sequences to several thousand, the groups are covered with different percentages. When aligning the sequence reads with blast (blastn with default parameters), the best hits were used in the taxonomy classification. Nevertheless, out of the ∼14,000 NCBI defined genera in the database, the primers covered ∼84% of them, which is in the range seen at the level of phyla (87%).

## Results

### 18S rRNA Gene Characterization

**Figure 1:**
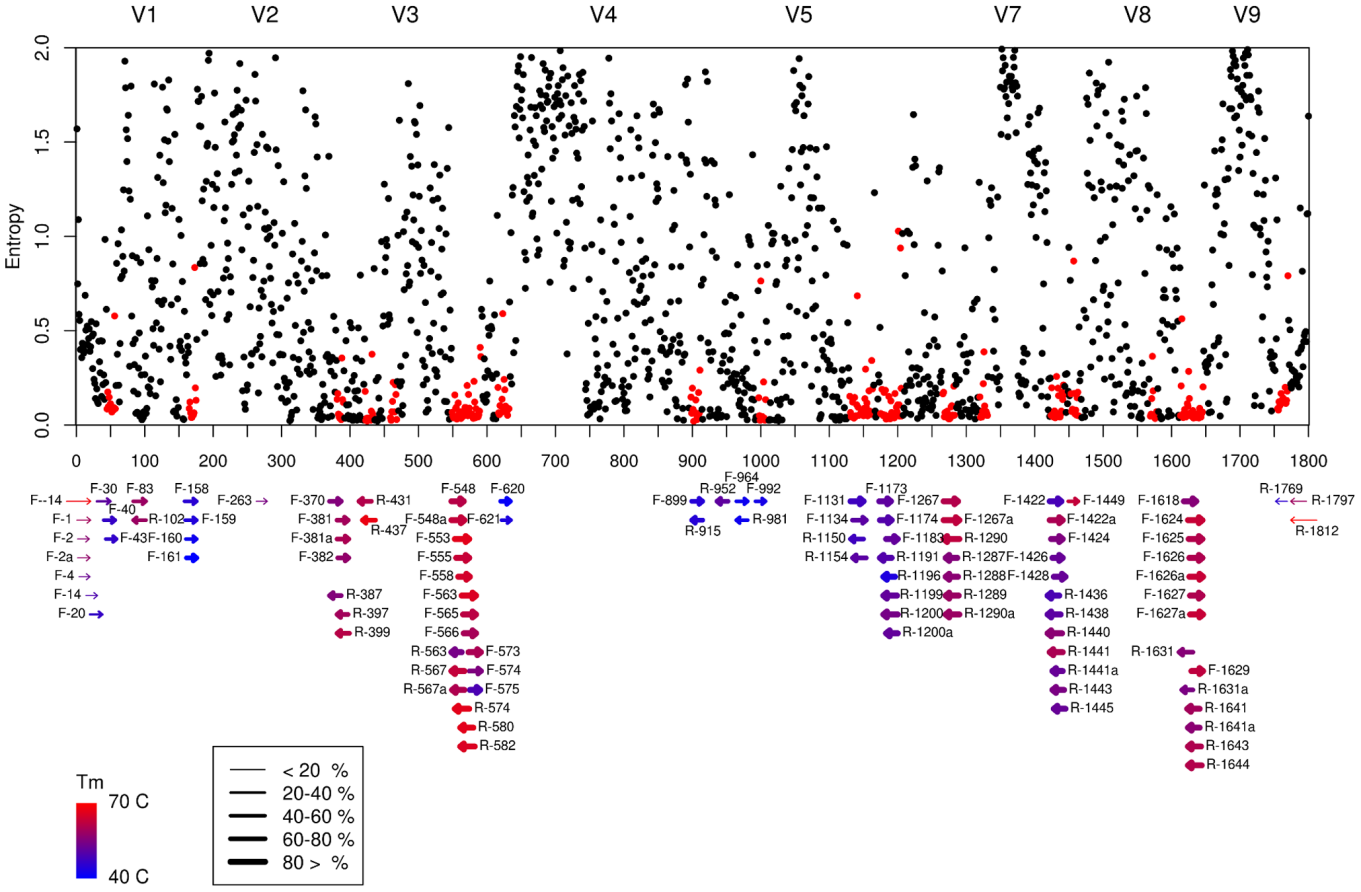
Nucleotide variability in 18S rRNA genes. Shannon entropy values of all eukaryotic alignment positions from the SILVA database along the 18S rRNA gene of *Saccharomyces cerevisiae.* Red dots mark consecutive nucleotide positions where at least 90% of ≥10 nt have entropy values lower than 0.2. The highly variable regions of 18S rRNA gene are denoted V1 to V9. In total, 100 primers targeting eukaryotes from the literature as well as those designed in this study are positioned along the reference sequence. The direction of the arrows indicates the orientation of the primers. The color denotes the melting temperature, and the thickness of the arrows represents the eukaryote universality of the primers.

To characterize the 18S rRNA gene, we used the aligned eukaryote sequences from the SILVA database. To describe the nucleotide variation in the alignment we calculated the Shannon entropy value of all nucleotide positions and mapped them to the *Saccharomyces cerevisiae* 18S rRNA gene as a reference (Figure 1). Low entropy values in the alignment position originated from a dominant nucleotide and should be interpreted as a conserved position. Regions of consecutive conserved positions are well suited for targeting the universal primers and were indicated in red in the plot (Figure 1). High entropy values on the other hand indicate a more uniform distribution of the 4 nucleotides in an alignment position. Long regions with high entropy will therefore represent higher taxonomic resolution and are more suitable as sequencing targets for biodiversity studies. The entropy values generated a structure of the 18S rRNA gene, with 8 characteristic variable regions flanked by conserved sequences. We used the standard nomenclature for the variable regions of the rRNA gene, V1–V9. However the eukaryote V6 region is relatively conserved and was therefore excluded (Figure 1). Even though the entropy values followed the known distribution of hypervariable regions, mapped conserved regions did not automatically determine the best region for universal primer targeting. The sites were simply used as a guide for suitable regions in the universal primer design process. Since different variable regions are not equal in length, their suitability as targets for biodiversity studies also depends on the sequencing length restrictions in the technology used. In this particular study, we based final primer selection to be optimized for the 454 pyrosequencing platform.

Each region was shown to have specific characteristics making it more or less suitable for biodiversity assessment using the chosen sequencing technology (Figure 1). The V1 region is rather short (ca 100 nt) and contains a highly conserved core segment. The V2 region on the other hand is longer (150–200 nt) with evenly distributed Shannon entropy. However, this region was neither highly variable, nor did it have any well-defined conserved segments suitable as target primer sites. The V3 region lacked highly variable segments and it was not very long. Although the V4 region has the highest length polymorphism [15], it is also the longest variable region in the rRNA gene (350 to 450 nt). A relatively conserved region of 70 nt is followed by a highly variable region of 120 nt, and additional short alterations between conserved and variable segments. The V5 is a short region without any long highly variable segments. V7 has a highly variable core of approximately 20–25 nt. The V8 region is over 150 nucleotides long with variable and conserved positions interspersed across the region with a conserved segment towards the 3′ end. The V9 was characterized by high nucleotide variability in the center of the region covering approximately 60 nt.

**Figure 2:**
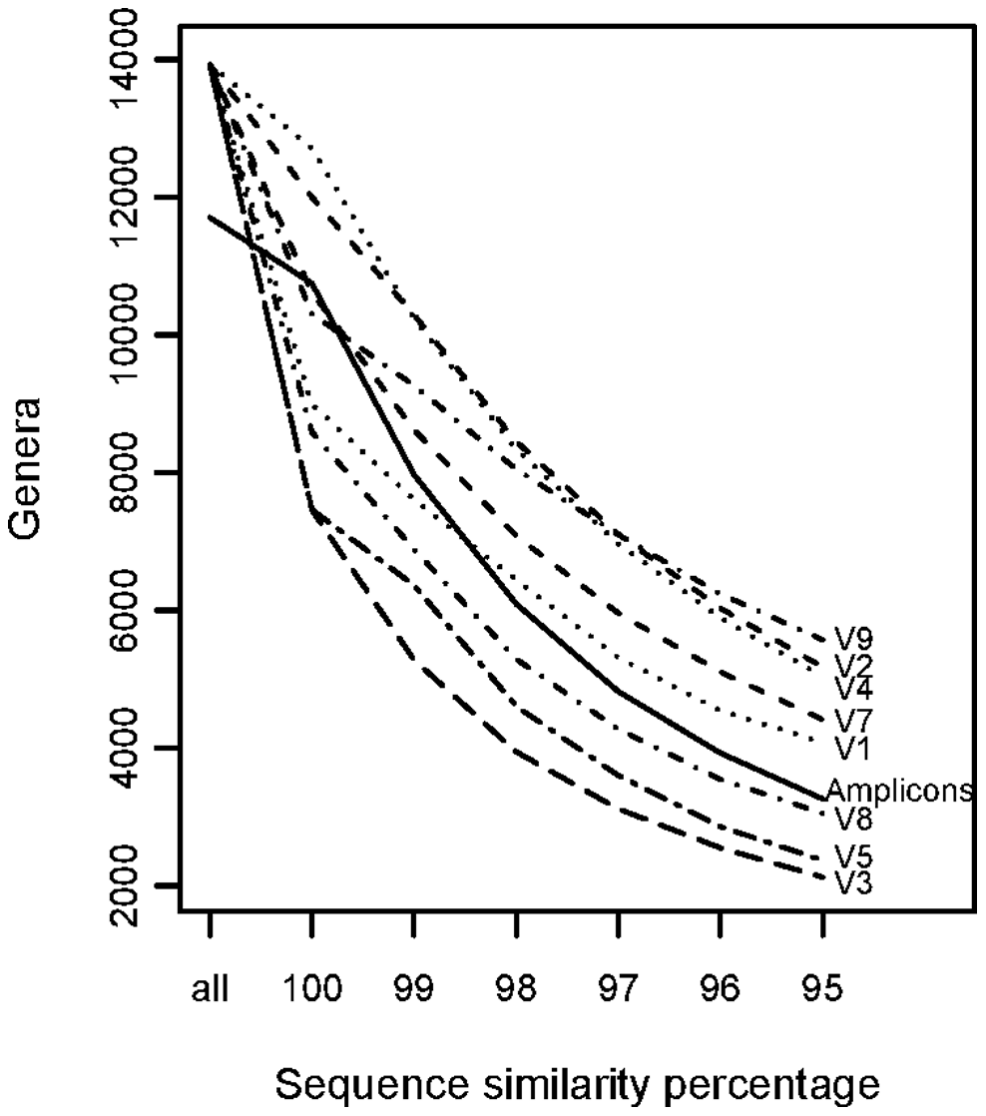
Variable regions of the 18S rRNA gene. The amplicon from the optimal primers selected in this study was included. Expected coverage of biodiversity is shown as a function of cut-off values for sequence similarity. V2, V4 and V9 generated the best results at all sequence identity cut-off levels.

To compare the applicability of the 8 variable regions of the SSU rRNA gene in diversity analyses, the nucleotide segments within each variable region from all eukaryote sequences in the database were clustered individually (Table S3). The number of distinguishable genera was plotted as the function of cut-off values (Figure 2). The slope of this regression enabled us to evaluate the taxonomic resolution (i.e. sequence diversity per length unit) of each variable region. The V2, V4 and V9 regions showed the highest variability, i.e. generated the largest number of genera as a function of clustering cut-off, and were therefore best suited for examining the biodiversity of a sample. These regions have been commonly used in many biodiversity studies [3]–[5], [41], [53]. The set of selected universal primers designed in this study was compared with respect to the variable regions targeted as well as the possibility of finding suitable primers (see criteria in methods).

### Eukaryote Specific Primers

Using the *de novo* primer design as well as primers found in the literature, 100 non-degenerate primers were identified as candidates for the optimal universal eukaryotic rRNA primer pair for biodiversity studies (see Table S1). Using the selection criteria described, 14 eukaryote specific primers, which were considered to be particularly well adapted for biodiversity studies, were selected from these 100 (eight forward and six reverse; listed in Table 1). These are flanking the V4, V5, V7 and V8 regions. However, out of the three regions most suitable to target biodiversity (i.e. V2, V4, V9), only the V4 region was represented by these selected primers, targeted by five (out of eight) forward and three (out of six) reverse primers (Table 1). Based on amplicon length, **Δ**T_m_ and the coverage of eukaryote groups, we propose the primer pair F-566∶5′ - CAG CAG CCG CGG TAA TTC C - 3′ and for R-1200∶5′ - CCC GTG TTG AGT CAA ATT AAG C - 3′. These primers amplify on average a 650 bp long fragment from the V4 and V5 regions, and were further tested in the laboratory for applicability in biodiversity studies.

**Table 1:** Primers generated following the *in silico* analysis.

Id	Sequence	Hits	Eukaryotes	Bacteria	Archaea	Tm	% GC
F-555	AAG TCT GGT GCC AGC AGC CG	50102	50102	0 (0/38)	0 (214/4667)	63	65
F-565	CCA GCA GCC GCG GTA ATT CC	49244	49244	0 (443/424948)	0 (104/7111)	61.5	65
F-566	CAG CAG CCG CGG TAA TTC C	49302	49302	0 (446/54367)	0 (130/12487)	59.4	63.2
F-573	CGC GGT AAT TCC AGC TCC A	46644	46644	0 (0/1)	0 (16/1158)	60	57.9
F-574	GCG GTA ATT CCA GCT CCA A	44206	44206	0 (0/0)	0 (0/17)	55.3	52.6
F-1183	AAT TTG ACT CAA CAC GGG	51566	51566	0 (0/1)	0 (0/12)	52	44.4
F-1422	ATA ACA GGT CTG TGA TGC	47935	47933	2 (2/2)	0 (0/0)	49.2	44.4
F-1624	CCT TTG TAC ACA CCG CCC GTC G	47314	47313	1 (31/1461)	0 (0/144)	62.7	63.6
R-574	CGG CTG CTG GCA CCA GAC TTG C	49862	49862	0 (0/0)	0 (0/0)	65.7	68.2
R-1196	TGT TGA GTC AAA TTA AGC	50044	50044	0 (1/42)	0 (0/152)	44.8	33.3
R-1200	CCC GTG TTG AGT CAA ATT AAG C	49503	49503	0 (0/0)	0 (0/0)	54.4	45.5
R-1289	ACT AAG AAC GGC CAT GCA CC	50601	50600	1 (1/1)	0 (0/0)	57.9	55
R-1438	CAT CAC AGA CCT GTT ATT GC	46673	46671	2 (2/2)	0 (0/0)	51.3	45
R-1631	TAC AAA GGG CAG GGA CG	44180	44179	1 (2/4)	0 (81/764)	54.6	58.8

The Id indicates the position of each primer with position references to the 18S rRNA gene of *Saccharomyces cerevisiae*. Hits indicate the total number of taxa that the primer pick up in the SILVA database, and categorized as eukaryote, bacteria or archaea hits. The numbers in parenthesis show results when allowing one and two mismatches. Tm refers to the estimated melting temperature and %GC indicates the percent of the nucleotides G and C in the primers using Integrated DNA Technologies.

### Optimal Primer Pair Characterization

An *in silico* PCR, without any mismatch allowed, using the primer pair (F-566– R-1200), covered 80% of all eukaryote sequences, with 87% coverage at the level of phyla in the SILVA database. No prokaryotes were amplified. The *in silico* results yielded amplification of 48 out of 56 known eukaryotic phyla included in the SILVA database (see Methods). The forward primer excluded *Haplosporidia* and *Lorificera*, while the reverse primer excluded *Acanthocephala*, *Cycliophora*, *Microsporidia* and *Myzostomida*. Both primers excluded *Rhombozoa* and *Rozella*. However, this might not accurately reflect the true diversity obtained when using the primers on environmental samples, because single nucleotide mismatches can still amplify some templates. Therefore, by allowing mismatches in the primer to template binding, we could obtain a more realistic understanding of the diversity covered by the primers. When one mismatch between the primers and template was allowed, the number of phyla covered by the *in silico* PCR was increased to 53, while still completely excluding prokaryotes. When two mismatches were allowed, we still excluded prokaryotes, while only *Rhomobozoa* and *Rozella* were excluded among the eukaryotic phyla. It is possible to use other combinations of the 8 selected primers (Table 1), which would include more phyla without any mismatch. For example, by exchanging the F-566 primer with F-555, we would also include the *Haplosporidia*. However, the melting properties of these two primers were very different (65°C vs. 54.4°C), with non-optimal PCR conditions as a consequence, which also makes the *in silico* predictions much less reliable.

**Figure 3:**
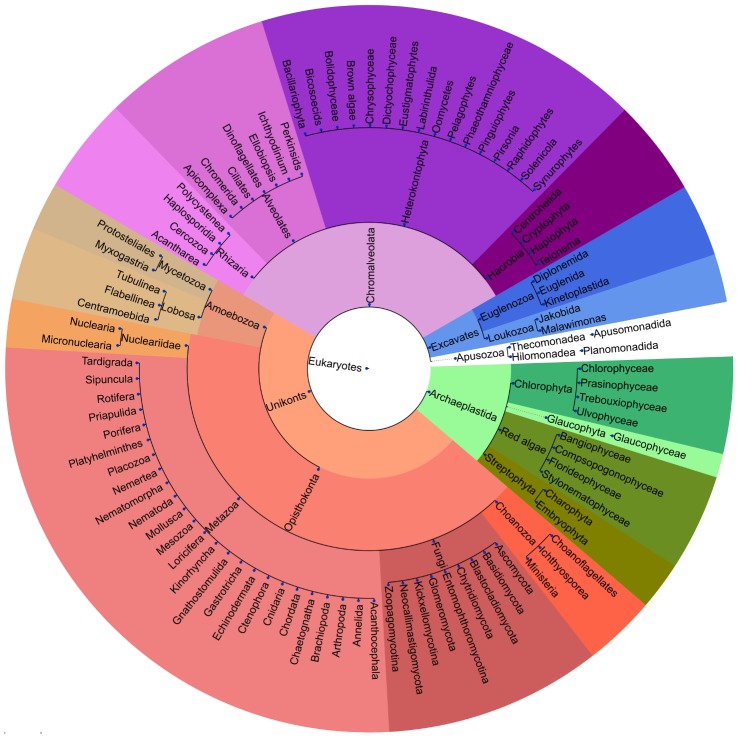
Empirical primer test on marine sediments. Taxonomic coverage of reads from 454 pyrosequencing on pooled DNA extracts from three different marine sediments from the Norwegian continental shelf. The figure indicates lower rank levels of the taxonomic tree of the best hits after running BLAST on representative sequences of 6823 OTUs against the SILVA database.

Although the *in silico* evaluation of the primers yielded full eukaryotic specificity there could be substantial differences in noise and resolution when applied *in situ*. The primers were therefore optimized and tested on three diverse, marine environmental sediment samples from the Norwegian continental shelf. As described in the Methods section, AmpliconNoise and QIIME post-sequencing pipelines were applied. Using default values for parameters to remove low quality reads, AmpliconNoise and QIIME produced 508,712 and 709,055 reads respectively. These pipelines further generated 6823 and 11129 OTUs in AmpliconNoise and QIIME, respectively. As predicted from the *in silico* analysis, the broad coverage of eukaryote taxonomic groups was confirmed *in situ*. The eukaryote groups identified using AmpliconNoise are represented in Figure 3. At the level of phyla, there was a 90% similarity between the two pipelines. The QIIME pipeline produced OTUs that were taxonomically mapped to an additional 8 eukaryote phyla (*Verdigellas*, *Rhodellophyceae*, *Voromonas*, *Xanthophyceae*, *Parabasalia*, *Trimastix*, *Priapulida* and *Sipuncula*), while AmpliconNoise produced 2 OTUs that were identified as *Chaetognatha* and *Gnathostomulida*. In addition, QIIME produced 5 OTUs that were identified as prokaryotes and 2 OTUs having ambiguous prokaryote/eukaryote identification. The QIIME-identified prokaryotes represent 0.06% of the total coverage, suggesting that the primers designed here can be considered as highly selective for eukaryotes in both pipelines tested. Indeed, QIIME generated a higher diversity of OTUs in our analysis. The discrepancy between the two pipelines could indicate that QIIME, without a denoising step, overestimated the diversity, or alternatively that AmpliconNoice was too conservative in estimating diversity. Interestingly, while neither *Rhombozoa* nor *Rozella* were found, as predicted by the *in silico* analysis, both AmpliconNoise and QIIME pipelines included OTUs from each of the phyla *Acanthocephala*, *Haplosporidia* and *Loricifera*. Representative sequences for the OTUs classified as the former two phyla yielded very low similarity to the SILVA and NCBI databases (10%), possibly indicating that the classification could be incorrect. The OTU classified as *Loricifera* on the other hand, was identified with a higher sequence similarity (89%) even though classification at the species level would be uncertain.

## Discussion

The overall aim of the study was to find the best possible set of universal eukaryote 18S rRNA primers, which could be used as standard primers in biodiversity studies. To achieve this goal, >50,000 eukaryotic sequences from the SILVA database were used for a characterization of the gene. This characterization allowed us to search for positions with high nucleotide conservation and carry out *de novo* primer design and evaluation. Even though the characterization of the 18S rRNA gene does not consider differences between eukaryote subgroups or between eukaryotes and prokaryotes, it gave us the opportunity to evaluate existing eukaryotic primers from the literature.

Mapping the SILVA base alignment (25,000 positions) to the *S. cerevisiae* 18S rRNA gene resulted in a 13-fold reduction in length of the alignment. However, the information that was removed consisted predominantly of gaps (Figure S1). The gaps were mostly observed at the regions close to 5′ and 3′ ends of the sequences. To our knowledge there is no biological explanation for this irregularity, and it could be the result of a bias in the alignment algorithm. We also observed higher nucleotide variation in the same regions, and primers targeting these areas subsequently had low coverage of eukaryote groups. Indeed, the information used when calculating the entropy values, as a proxy for nucleotide variation, is dependent on the correctness of the alignment. However, improving the alignment of eukaryotes in the SILVA database was not an objective of this study.

Mapping of the primer candidates to the 18S rRNA alignment yielded correlation between the conserved regions and primer target sites (Figure 1). Indeed, the mapped positions of the highly variable and conserved regions were consistent with previously published characterizations [9], [10]. The best primer pair amplified a 650 bp fragment of the V4 and V5 region of the 18S rRNA gene. Both *in silico* tests and a test on marine sediments using 454 pyrosequencing indicated that no prokaryotic sequences were amplified. These results suggest that the primers are well suited for eukaryotic biodiversity analysis as the full potential of the high throughput sequencing platform could be used. However, our *in silico* analysis indicated that the primers were not fully universal for eukaryotes, as they generated reads from 48 out of 56 NCBI phyla present in the SILVA database. Indeed, these primers yield considerably lower variability, and in consequence, lower biodiversity descriptive value, than the full potential of the variable region (Figure 2).

Eight phyla could not be amplified using the optimal primer pair proposed here (*Acanthocephala, Cycliophora, Haplosporidia, Loricifera, Microsporidia, Myzostomida, Rhombozoa* and *Rozella*). However, both *Cycliophora* and *Lorificera* were represented by relatively few sequences in the SILVA database, and the *in silico* analysis might therefore not accurately reflect these phyla. On the other hand, the inclusion of some groups was also based on a limited number of sequence entries in the SILVA database. Nevertheless, high throughput sequencing of sediment samples demonstrated that the primers generated in this study did pick up three of the phyla for which the *in silico* analysis predicted exclusion. It is not clear why there were discrepancies between the *in silico* and the *in situ* results regarding these three phyla, but it is possible that the relevant groups were insufficiently represented in the SILVA database. On the other hand, the primers may have amplified the sequences with mismatches in the PCR because the position of each mismatch is also important. Indeed, our *in silico* tests show that the primers successfully amplified the groups with only one (*Haplosporidia*, *Loricifera*) or two (*Acantocephala*) mismatches. These results also demonstrate the trade-off between exclusion of some groups (i.e. prokaryotes) and the universality of another group (i.e. eukaryotes). The results from the empirical test regarding the exclusion of prokaryotes were fully consistent with the *in silico* analysis. The coverage of the target groups in the two tests was also highly consistent. Although prokaryotic sequences could subsequently be filtered out before or during the analysis they would reduce the amount of useful sequence data to be subsequently analyzed, hence also reducing the descriptive value.

In the SILVA database, some taxonomic groups were represented by few sequences, while other groups had a comprehensive representation. The *in silico* analysis could therefore be sensitive to either false positive or false negative conclusions, leading to evaluation errors in estimated primer coverage. A relatively large number of microorganisms from sampling projects cannot be cultured and clearly identified, and many of these sequences have been omitted from the SILVA database or are present with identities of poor certainty. For the purpose of the current evaluation however, the sequence alignment in SILVA at the rank of kingdom was sufficient, and for this reason we did not remove these organisms from the alignment and further analyses.

Prior to choosing an optimal universal eukaryotic primer set for high throughput sequencing, we analyzed the position specific nucleotide variation as a proxy for taxonomic resolution of all the 8 variable regions. The analysis indicated that V2, V4 and V9 yielded the highest taxonomic resolution at cut-off values ranging from 95–100% sequence identity (Figure 2). However, when implementing the criteria for optimal primer choice only the V4 region could be effectively amplified (Table 1). There is an ongoing debate as to which of the variable 18S rRNA regions is most applicable for biodiversity studies [3]–[5], [18]. This study demonstrates that the most applicable region should also be assessed considering available primer sites for a given region. The characterization of the 18S rRNA gene indicates that there were no nucleotide segments of sufficient length for standard PCR along the whole gene that were entirely conserved within all eukaryotes, while being absent in prokaryotes. This means that one cannot design a single primer pair that will cover the full eukaryotic diversity and at the same time exclude prokaryotes. The primers designed in this study covered 80% of all eukaryotes present in databases. This fraction could be improved by increasing degeneracy in the primers, a common strategy in designing universal primers with high coverage. However, increased degeneracy reduces specificity and can lead to amplification of non-targeted taxa in the PCR assay hence reducing resolution in the final product. Furthermore it introduces bias in template-to-product ratios [54] and an increased divergence between the results *in silico* and *in situ*. From rigorous analyses of previously published primers (Table S2) and an extensive effort in designing new primers, our results suggests that the F-566 and R-1200 were the best universal eukaryotic primers among those tested for general biodiversity studies using 454 pyrosequencing. The amplicons generated using these primers averaged 650 nt in length and covered the variable regions V4 and V5. Even though the applied 454 pyrosequencing technology does not generate reads of the full 650 nt length, the ∼400 nt reads will cover the V4 region, which is also one of the most variable 18S rRNA regions (Figure 1). Other sequencing platforms, such as Ion Torrent [55] or Illumina [56], currently have limitations of amplicon size. The characterization of the 18S rRNA gene in this study can also be used to search for optimal primer pairs for these technologies. Our *in silico* analyses suggests that the primer pair F-574 and R-952 or F-1183 and R-1631a could be used to produce shorter amplicons covering the V4 region or V7/V8 region respectively, without including prokaryotes (with up to 2 mismatches) (Figure 1 and Table S1). However, the coverage of eukaryote taxa may not be as good as with the universal eukaryotic primers suggested by this study. In general, there will be a loss of taxonomic resolution when using sequencing platforms that generate shorter sequence read information. Therefore, for those metagenomic studies in which the highest levels of taxonomic resolution are desired, there will be a preference for sequencing platforms generating, or capable of generating in the near future, longer sequence reads.

Significant length polymorphisms will have an effect on primer selection, and the 18S rRNA gene does have variable length. Our *in silico* analysis did predict distribution of the lengths of the resulting amplicons across different taxa ranging from 318 to 1800 (Figure S2). However, 90% of the amplicons were predicted to be in the range 530–700 nt and 80% of the predicted amplicons were in the range 600–650 nt. This suggests insertions/deletions across the taxa. Nevertheless, a large proportion of the taxa in the *in silico* analysis did fulfill the length criteria of the 454 pyrosequencing platform. Our data further indicated that the majority of amplicons in the lower range (530–600 nt) were dominated by alveolates and fungi, whereas arthropods (hexapods) dominated the upper range (650–700 nt).

High throughput sequencing shows great promise in ecological studies and biomonitoring programs. Not only does it allow characterization of microbial communities quickly and at low cost, but it has also helped to record the presence of organisms that have been difficult to culture. However, there are important challenges tied to the technology such as experimental biases and differences that make studies difficult to compare. Sampling biases have received attention in ecological studies, more extensively in prokaryotes than in eukaryotes [53]. The need for identifying and removing sequencing and PCR errors, which otherwise could result in an overestimated biodiversity, has also been addressed by several algorithms that have become an important step in the analysis of amplicon sequence data. However, the choice of universal eukaryote primers targeting the 18S rRNA gene has not been adequately evaluated previously. In this study we have shown that it is possible to design universal eukaryote primers with wide coverage of eukaryote groups and without resorting to high degeneracy. The proposed optimal primers were also demonstrated to produce amplicon sequence with good resolution when applied to environmental samples from marine sediments, while completely excluding non-targeted prokaryotes.

## Supporting Information

Figure S1 **Estimated information loss from the excluded alignment positions.** The figure shows the percentage of sequences in the database (y-axis) having a nucleotide in the excluded alignment positions. As the number of excluded positions increases, the information content in them drops significantly.
(DOCX)

Figure S2 **Length distribution of amplicons produced by the chosen primer set from** ***in silico*** **analysis.**
(DOCX)

Table S1 **Unique non-degenerate universal eukaryotic primers tested in Silva v.106.** Primer ID indicates orientation and position of the primer. Melting temperature and GC percentage were calculated using Integrated DNA Technologies.
(DOCX)

Table S2 **Eukaryote specific primers from previouse publications.** The primers were used as a start when searching for optimal primers in the biodiversity assessment experiment.
(DOCX)

Table S3 **Start and end positions in the 18S rRNA gene, which was used to define the variable regions in** **Figure 2** **.**
(DOCX)
