# Supplementary material for: Characterization of the 18S rRNA Gene for Designing Universal Eukaryote Specific Primers
Source: PLoS One. 2014 Feb 7;9(2):e87624. doi: 10.1371/journal.pone.0087624 (PMC3917833; doi:10.1371/journal.pone.0087624)

Figure S1. Estimated information loss from the excluded alignment positions. The figure shows the percentage of sequences in the database (y-axis) having a nucleotide in the excluded alignment positions. As the number of excluded positions increases, the information content in them drops significantly.


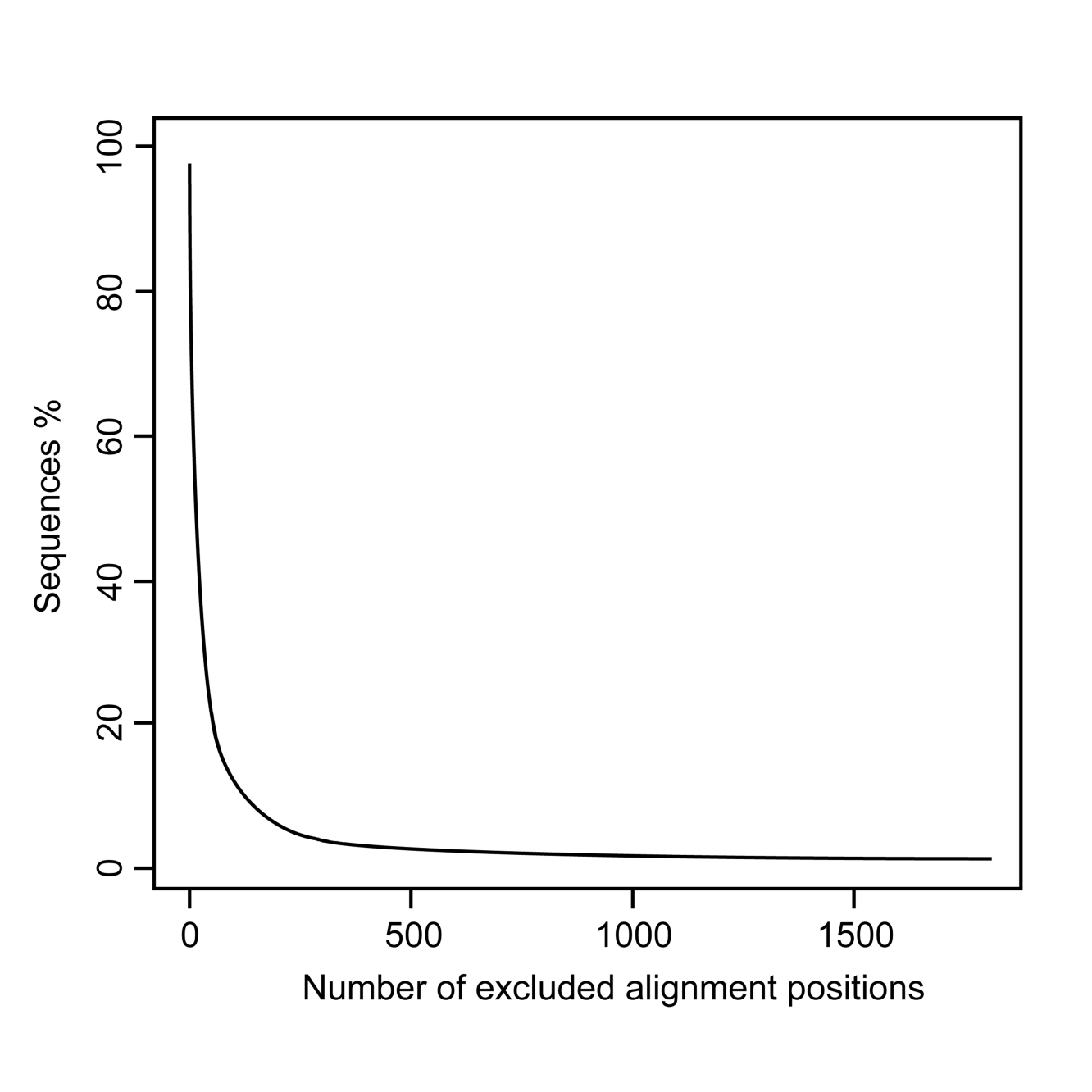

Supplement: Figure S1 — Estimated information loss from the excluded alignment positions. The figure shows the percentage of sequences in the database (y-axis) having a nucleotide in the excluded alignment positions. As the number of excluded positions increases, the information content in them drops significantly. (DOCX) [file pone.0087624.s001.docx]
