# Supplementary material for: Characterization of the 18S rRNA Gene for Designing Universal Eukaryote Specific Primers
Source: PLoS One. 2014 Feb 7;9(2):e87624. doi: 10.1371/journal.pone.0087624 (PMC3917833; doi:10.1371/journal.pone.0087624)

Figure S2. Length distribution of amplicons produced by the chosen primer set from *in silico* analysis.


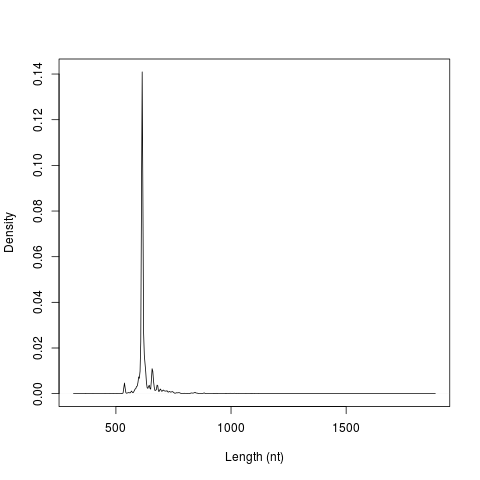

Supplement: Figure S2 — Length distribution of amplicons produced by the chosen primer set from in silico analysis. (DOCX) [file pone.0087624.s002.docx]
