# Supplementary material for: Characterization of the 18S rRNA Gene for Designing Universal Eukaryote Specific Primers
Source: PLoS One. 2014 Feb 7;9(2):e87624. doi: 10.1371/journal.pone.0087624 (PMC3917833; doi:10.1371/journal.pone.0087624)
Supplement: Table S1 — Unique non-degenerate universal eukaryotic primers tested in Silva v.106. Primer ID indicates orientation and position of the primer. Melting temperature and GC percentage were calculated using Integrated DNA Technologies. (DOCX) [file pone.0087624.s003.docx]

Table S1. Unique non-degenerate universal eukaryotic primers tested in Silva v.106. Primer ID indicates orientation and position of the primer. Melting temperature and GC percentage were calculated using Integrated DNA Technologies.

| Id | Sequence | Hits | Eukaryotes | Bacteria | Archaea | Tm | GC % |
| --- | --- | --- | --- | --- | --- | --- | --- |
| F–14 [34] | CCG AAT TCG TCG ACA ACC TGG TTG ATC CTG CCA GT | 39 | 39 | 0 | 0 | 67.5 | 54.3 |
| F-1 [35] | AAC CTG GTT GAT CCT GCC AGT | 3157 | 3155 | 0 | 2 | 58.9 | 52.4 |
| F-2 | ACC TGG TTG ATC CTG CCA | 6663 | 6657 | 0 | 6 | 56.5 | 55.6 |
| F-2a | ACC TGG TTG ATC CTG CCA G | 6636 | 6631 | 0 | 5 | 57.3 | 57.9 |
| F-4 | CTG GTT GAT CCT GCC AG | 10848 | 10515 | 0 | 333 | 52.9 | 58.8 |
| F-14 [40] | CTG CCA GTA GTC ATA TGC | 10902 | 10902 | 0 | 0 | 49.9 | 50 |
| F-20 | GTA GTC ATA TGC TTG TCT C | 12215 | 12215 | 0 | 0 | 47.2 | 42.1 |
| F-30 [41] | GCT TGT CTC AAA GAT TAA GCC | 20064 | 20064 | 0 | 0 | 51.7 | 42.9 |
| F-40 | AAG ATT AAG CCA TGC ATG | 27212 | 27212 | 0 | 0 | 47.6 | 38.9 |
| F-43 | ATT AAG CCA TGC ATG TC | 23332 | 23331 | 0 | 1 | 47.1 | 41.2 |
| F-83 [42] | GAA ACT GCG AAT GGC TCA TT | 23018 | 23018 | 0 | 0 | 58 | 45 |
| F-158 | TTC TAG AGC TAA TAC ATG C | 30725 | 30725 | 0 | 0 | 46 | 36.8 |
| F-159 | TCT AGA GCT AAT ACA TGC | 31841 | 31841 | 0 | 0 | 45.2 | 38.9 |
| F-160 | CTA GAG CTA ATA CAT GC | 32828 | 32828 | 0 | 0 | 43.2 | 41.2 |
| F-161 | TAG AGC TAA TAC ATG C | 33048 | 33048 | 0 | 0 | 41.2 | 37.5 |
| F-263 [35] | CGA ATC GCA TGG CCT TG | 7616 | 7616 | 0 | 0 | 54.5 | 58.8 |
| F-370 | AGG GTT CGA TTC CGG AGA | 38090 | 38090 | 0 | 0 | 55.4 | 55.6 |
| F-381 | CCG GAG AGG GAG CCT GA | 42778 | 42778 | 0 | 0 | 59.1 | 70.6 |
| F-381a | CCG GAG AGG GAG CCT GAG | 42570 | 42570 | 0 | 0 | 59.8 | 72.2 |
| F-382 | CGG AGA GGG AGC CTG AG | 43285 | 43285 | 0 | 0 | 57.2 | 70.6 |
| F-548 | GGA GGG CAA GTC TGG TGC C | 47247 | 47247 | 0 | 0 | 61.2 | 68.4 |
| F-548a | GGA GGG CAA GTC TGG TGC CA | 47176 | 47176 | 0 | 0 | 62.5 | 65 |
| F-553 | GCA AGT CTG GTG CCA GCA GCC G | 49862 | 49862 | 0 | 0 | 65.7 | 68.2 |
| F-555 | AAG TCT GGT GCC AGC AGC CG | 50102 | 50102 | 0 | 0 | 63 | 65 |
| F-558 | TCT GGT GCC AGC AGC CGC | 50448 | 50448 | 0 | 0 | 64.2 | 72.2 |
| F-563 | TGC CAG CAG CCG CGG TAA TTC C | 48953 | 48953 | 0 | 0 | 64.9 | 63.6 |
| F-565 | CCA GCA GCC GCG GTA ATT CC | 49244 | 49244 | 0 | 0 | 61.5 | 65 |
| F-566 | CAG CAG CCG CGG TAA TTC C | 49302 | 49302 | 0 | 0 | 59.4 | 63.2 |
| F-573 [42] | CGC GGT AAT TCC AGC TCC A | 46644 | 46644 | 0 | 0 | 60 | 57.9 |
| F-574 | GCG GTA ATT CCA GCT CCA A | 44206 | 44206 | 0 | 0 | 55.3 | 52.6 |
| F-575 | CGG TAA TTC CAG CTC C | 47842 | 47742 | 0 | 100 | 49.5 | 56.3 |
| F-620 | AAA AGC TCG TAG TTG | 42060 | 42060 | 0 | 0 | 42.2 | 40 |
| F-621 | AAA GCT CGT AGT TGG | 28072 | 28072 | 0 | 0 | 44.9 | 46.7 |
| F-899 | GAG GTG AAA TTC TTG GA | 43442 | 43442 | 0 | 0 | 45.9 | 41.2 |
| F-964 | TTA ATC AAG AAC GAA AGT | 29904 | 29904 | 0 | 0 | 43.2 | 27.8 |
| F-992 | AAG ACG ATC AGA TAC C | 26595 | 26595 | 0 | 0 | 44 | 43.8 |
| F-1131 | AAA CTT AAA GGA ATT GAC GG | 46059 | 43519 | 2398 | 142 | 48.3 | 35 |
| F-1134 | CTT AAA GGA ATT GAC GGA AGG | 42040 | 42040 | 0 | 0 | 51.2 | 42.9 |
| F-1173 | CCT GCG GCT TAA TTT GAC | 46834 | 46834 | 0 | 0 | 51.6 | 50 |
| F-1174 | CTG CGG CTT AAT TTG ACT | 47417 | 47417 | 0 | 0 | 50.3 | 44.4 |
| F-1183 [42] | AAT TTG ACT CAA CAC GGG | 51566 | 51566 | 0 | 0 | 52 | 44.4 |
| F-1267 | GGT GGT GCA TGG CCG TTC TTA G | 49719 | 49718 | 1 | 0 | 60.7 | 59.1 |
| F-1267a | GGT GGT GCA TGG CCG TTC TTA GTT | 49535 | 49534 | 1 | 0 | 61.8 | 54.2 |
| F-1422 | ATA ACA GGT CTG TGA TGC | 47935 | 47933 | 2 | 0 | 49.2 | 44.4 |
| F-1422a [42] | ATA ACA GGT CTG TGA TGC CC | 47730 | 47728 | 2 | 0 | 54.6 | 50 |
| F-1424 | AAC AGG TCT GTG ATG CCC | 48165 | 48163 | 2 | 0 | 55 | 55.6 |
| F-1426 | CAG GTC TGT GAT GCC C | 48404 | 48402 | 2 | 0 | 52.7 | 62.5 |
| F-1428 | GGT CTG TGA TGC CCT TAG | 46836 | 46834 | 2 | 0 | 52.4 | 55.6 |
| F-1449 | TTC TGG GCC GCA CGC G | 28463 | 28452 | 1 | 10 | 62.6 | 75 |
| F-1618 | CCC TGC CCT TTG TAC ACA C | 47653 | 47649 | 2 | 2 | 56.1 | 57.9 |
| F-1624 | CCT TTG TAC ACA CCG CCC GTC G | 47314 | 47313 | 1 | 0 | 62.7 | 63.6 |
| F-1625 | CTT TGT ACA CAC CGC CCG TCG | 47498 | 47496 | 2 | 0 | 60.8 | 61.9 |
| F-1626 | TTT GTA CAC ACC GCC CGT CG | 48917 | 48897 | 10 | 10 | 60.3 | 60 |
| F-1626a | TTT GTA CAC ACC GCC CGT CGC | 47737 | 47730 | 7 | 0 | 62.8 | 61.9 |
| F-1627 | TTG TAC ACA CCG CCC GTC G | 49713 | 49287 | 411 | 15 | 60.4 | 63.2 |
| F-1627a | TTG TAC ACA CCG CCC GTC GC | 48434 | 48106 | 327 | 1 | 62.9 | 65 |
| F-1629 | GTA CAC ACC GCC CGT CGC | 48598 | 48270 | 327 | 1 | 61.9 | 72.2 |
| R-102 | AAT GAG CCA TTC GCA GTT TC | 23018 | 23018 | 0 | 0 | 58 | 45 |
| R-387 | TCT CCG GAA TCG AAC CCT | 38090 | 38090 | 0 | 0 | 55.4 | 55.6 |
| R-397 [48] | TCA GGC TCC CTC TCC GG | 42778 | 42778 | 0 | 0 | 59.1 | 70.6 |
| R-399 | TCT CAG GCT CCC TCT CCG G | 42522 | 42522 | 0 | 0 | 60.9 | 68.4 |
| R-431 | GCC TGC TGC CTT CCT TGG A | 38902 | 38902 | 0 | 0 | 60.6 | 63.2 |
| R-437 | TTG CGC GCC TGC TGC CTT CC | 38093 | 38093 | 0 | 0 | 66.5 | 70 |
| R-563 [38] | ACC AGA CTT GCC CTC C | 47959 | 47540 | 0 | 419 | 54.3 | 62.5 |
| R-567 | TGG CAC CAG ACT TGC CCT CC | 47176 | 47176 | 0 | 0 | 62.5 | 65 |
| R-567a | TGG CAC CAG ACT TGC CCT C | 50546 | 50543 | 0 | 3 | 60.3 | 63.2 |
| R-574 | CGG CTG CTG GCA CCA GAC TTG C | 49862 | 49862 | 0 | 0 | 65.7 | 68.2 |
| R-580 | TTA CCG CGG CTG CTG GCA CC | 51316 | 50907 | 98 | 311 | 65.8 | 70 |
| R-582 | AAT TAC CGC GGC TGC TGG CAC C | 48939 | 48939 | 0 | 0 | 65.1 | 63.6 |
| R-915 | TCC AAG AAT TTC ACC TC | 43442 | 42442 | 0 | 0 | 45.9 | 41.2 |
| R-952 | TTG GCA AAT GCT TTC GC | 37459 | 37459 | 0 | 0 | 51.9 | 47.1 |
| R-981 | ACT TTC GTT CTT GAT TAA | 29904 | 29904 | 0 | 0 | 43.2 | 27.8 |
| R-1150 | CCG TCA ATT CCT TTA AGT TT | 46059 | 43519 | 2398 | 142 | 48.3 | 35 |
| R-1154 | CCT TCC GTC AAT TCC TTT AAG | 42040 | 42040 | 0 | 0 | 51.2 | 42.9 |
| R-1191 | AGT CAA ATT AAG CCG CAG | 47417 | 47417 | 0 | 0 | 50.3 | 44.4 |
| R-1196 | TGT TGA GTC AAA TTA AGC | 50044 | 50044 | 0 | 0 | 44.8 | 33.3 |
| R-1199 | CCG TGT TGA GTC AAA TTA AGC | 49593 | 49593 | 0 | 0 | 52.2 | 42.9 |
| R-1200 | CCC GTG TTG AGT CAA ATT AAG C | 49503 | 49503 | 0 | 0 | 54.4 | 45.5 |
| R-1200a | CCC GTG TTG AGT CAA ATT | 51566 | 51566 | 0 | 0 | 52 | 44.4 |
| R-1287 | TAA GAA CGG CCA TGC ACC | 50828 | 50827 | 1 | 0 | 55.6 | 55.6 |
| R-1288 | CTA AGA ACG GCC ATG CAC C | 50680 | 50679 | 1 | 0 | 56.4 | 57.9 |
| R-1289 | ACT AAG AAC GGC CAT GCA CC | 50601 | 50600 | 1 | 0 | 57.9 | 55 |
| R-1290 | AAC TAA GAA CGG CCA TGC ACC ACC | 49535 | 49534 | 1 | 0 | 61.8 | 54.2 |
| R-1290a | AAC TAA GAA CGG CCA TGC ACC | 50490 | 50489 | 1 | 0 | 58 | 52.4 |
| R-1436 | TCA CAG ACC TGT TAT TGC | 46783 | 46781 | 2 | 0 | 49.4 | 44.4 |
| R-1438 | CAT CAC AGA CCT GTT ATT GC | 46673 | 46671 | 2 | 0 | 51.3 | 45 |
| R-1440 | GGC ATC ACA GAC CTG TTA TTG C | 46559 | 46557 | 2 | 0 | 56.4 | 50 |
| R-1441 [42] | GGG CAT CAC AGA CCT GTT AT | 47730 | 47728 | 2 | 0 | 60 | 50 |
| R-1441a | GGG CAT CAC AGA CCT G | 48404 | 48402 | 2 | 0 | 52.7 | 62.5 |
| R-1443 | AAG GGC ATC ACA GAC CTG | 47658 | 47656 | 2 | 0 | 54.7 | 55.6 |
| R-1445 [40] | CTA AGG GCA TCA CAG ACC | 46836 | 46834 | 2 | 0 | 52.4 | 55.6 |
| R-1631 [41] | TAC AAA GGG CAG GGA CGT AAT | 36248 | 36248 | 0 | 0 | 56.1 | 47.6 |
| R-1631a | TAC AAA GGG CAG GGA CG | 44180 | 44179 | 1 | 0 | 54.6 | 58.8 |
| R-1641 | GGG CGG TGT GTA CAA AGG G | 47526 | 47523 | 3 | 0 | 58.8 | 63.2 |
| R-1641a | GGG CGG TGT GTA CAA AGG | 47892 | 47860 | 32 | 0 | 56.4 | 61.1 |
| R-1643 | ACG GGC GGT GTG TAC AAA GG | 47538 | 47509 | 29 | 0 | 60.3 | 60 |
| R-1644 | GAC GGG CGG TGT GTA CAA AGG | 47482 | 47453 | 29 | 0 | 60.7 | 61.9 |
| R-1769 | ACC TTG TTA CGA CTT TAC | 763 | 42 | 720 | 1 | 46.4 | 38.9 |
| R-1797 | TGA TCC TTC TGC AGG TTC ACC TAC | 3549 | 3547 | 0 | 2 | 58.4 | 50 |
| R-1812 [34] | CCC GGG ATC CAA GCT TGA TCC TTC TGC AGG TTC ACC TAC | 114 | 114 | 0 | 0 | 68.6 | 56.4 |
