# Supplementary material for: Characterization of the 18S rRNA Gene for Designing Universal Eukaryote Specific Primers
Source: PLoS One. 2014 Feb 7;9(2):e87624. doi: 10.1371/journal.pone.0087624 (PMC3917833; doi:10.1371/journal.pone.0087624)
Supplement: Table S2 — Eukaryote specific primers from previouse publications. The primers were used as a start when searching for optimal primers in the biodiversity assessment experiment. (DOCX) [file pone.0087624.s004.docx]

Table S2. Eukaryote specific primers from previouse publications. The primers were used as a start when searching for optimal primers in the biodiversity assessment experiment.

| Direction | Sequence | Degeneracy | Publication |
| --- | --- | --- | --- |
| F | CCG AAT TCG TCG ACA ACC TGG TTG ATC CTG CCA GT | 1 | Medlin, 1988 [34] |
| F | AAC CTG GTT GAT CCT GCC AGT | 1 | Jebaraj, 2010 [35] |
| F | ACC TGG TTG ATC CTG CCA | 1 | Romari, 2004 [36] |
| F | ACC TGG TTG ATC CTG CCA G | 1 | Moon-van Der Staay, 2000 [37] |
| F | CTG GTT GAT CCT GCC AG | 1 | Diez, 2001 [38] |
| F | CTG GTT GAT YCT GCC AGT | 2 | Lovejoy, 2010 [39] |
| F | CTG CCA GTA GTC ATA TGC | 1 | Troedsson, 2008 [40] |
| F | GTA GTC ATA TGC TTG TCT C | 1 | Jebaraj, 2010 [35] |
| F | GCT TGT CTC AAA GAT TAA GCC | 1 | Creer, 2010 [41] |
| F | GAA ACT GCG AAT GGC TCA TT | 1 | Winnepenninckx, 1994 [42] |
| F | CGA ATC GCA TGG CCT TG | 1 | Jebaraj, 2010 [35] |
| F | AGG GYT CGA YYC CGG AGA | 8 | Winnepenninckx,1994 [42] |
| F | CTG GTG CCA GCA GCC GCG GCA A | 2 | Machida 2012 [50] |
| F | GTG CCA GCM GCC GCG G | 2 | Edgcomb, 2002 [43] |
| F | CCA GCA SCY GCG GTA ATT CC | 4 | Stoeck, 2010 [44] |
| F | CGC GGT AAT TCC AGC TCC A | 1 | Winnepenninckx, 1994 [42] |
| F | GCG GTA ATT CCA GCT CCA A | 1 | Lovejoy, 2010 [39] |
| F | CGG TAA TTC CAG CTC C | 1 | Stoeck, 2006 [44] |
| F | GAG GTG AAA TTC TTR GA | 2 | Troedsson, 2008 [40] |
| F | TTR ATC AAG AAC GAA AGT | 2 | Winnepenninckx, 1994 [42] |
| F | AAR AYG ATY AGA TAC C | 8 | Hendriks, 1991 [46] |
| F | AAA CTY AAA GRA ATT GAC GG | 4 | Troedsson, 2008 [40] |
| F | AAT TTG ACT CAA CAC GGG | 1 | Winnepenninckx, 1994 [42] |
| F | GGT GGT GCA TGG CCG TTC TTA GTT | 1 | Creer, 2010 [41] |
| F | ATA ACA GGT CTG TGA TGC CC | 1 | Winnepenninckx, 1994 [42] |
| F | CAG GTC TGT GAT GCC C | 1 | Diez, 2001 [38] |
| F | CCC TGC CHT TTG TAC ACA C | 3 | Amaral-Zettler, 2009 [47] |
| F | TTT GYA CAC ACC GCC CGT CG | 2 | Winnepenninckx, 1994 [42] |
| F | TTG TAC ACA CCG CCC | 1 | Amaral-Zettler, 2009 [47] |
| F | GTA CAC ACC GCC CGT C | 1 | Stoeck, 2010 [51] |
| R | AAT GAG CCA TTC GCA GTT TC | 1 | Winnepenninckx, 1994 [42] |
| R | TCT CCG GRR TCG ARC CCT | 8 | Winnepenninckx, 1994 [42] |
| R | TCA GGC TCC CTC TCC GG | 1 | Lim, 1993 [48] |
| R | TCT CAG GCT CCY TCT CCG G | 2 | Winnepenninckx, 1994 [42] |
| R | GCC TGC TGC CTT CCT TGG A | 1 | Creer, 2010 [41] |
| R | ACC AGA CTT GCC CTC C | 1 | Diez, 2001 [38] |
| R | ATT ACC GCG GCT GCT GGC | 1 | Winnepenninckx, 1994 [42] |
| R | TTA CCG CGG CTG CTG G | 1 | Troedsson, 2008 [40] |
| R | TTG GYR AAT GCT TTC GC | 4 | Winnepenninckx, 1994 [42] |
| R | ACT TTC GTT CTT GAT YRA | 4 | Stoeck, 2010 [51] |
| R | CCG TCA ATT YYT TTR AGT TT | 8 | Winnepenninckx, 1994 [42] |
| R | AGT CAA ATT AAG CCG CAG | 1 | Jebaraj, 2010 [35] |
| R | GGG CAT CAC AGA CCT GTT AT | 1 | Winnepenninckx, 1994 [42] |
| R | GGG CAT CAC AGA CCT G | 1 | Lim, 1993 [48] |
| R | CTA AGG GCA TCA CAG ACC | 1 | Troedsson, 2008 [40] |
| R | TAC AAA GGG CAG GGA CGT AAT | 1 | Creer, 2010 [41] |
| R | GGG CGG TGT GTA CAA RGR G | 4 | Stoeck, 2006 [44] |
| R | ACG GGC GGT GTG TRC | 2 | Diez, 2001 [38] |
| R | GAC GGG CGG TGT GTR C | 2 | Troedsson, 2008 [40] |
| R | AIC CAT TCA ATC GGT AIT | 9 | Jebaraj, 2010 [35] |
| R | ANC CAT TCA ATC GGT ANT | 16 | Jebaraj, 2010 [35] |
| R | ACC TTG TTA CGA CTT TAC | 1 | Gruebl, 2002 [49] |
| R | ACC TTG TTA CGR CTT | 2 | Stoeck, 2006 [44] |
| R | CYG CAG GTT CAC CTA CRG | 4 | Winnepenninckx, 1994 [42] |
| R | TGA TCC TTC TGC AGG TTC ACC TAC | 1 | Lovejoy, 2010 [39] |
| R | CCC GGG ATC CAA GCT TGA TCC TTC TGC AGG TTC ACC TAC | 1 | Medlin, 1988 [34] |
| R | TGA TCC TTC YGC AGG TTC AC | 2 | Romari, 2004 [36] |
