# Supplementary material for: Characterization of the 18S rRNA Gene for Designing Universal Eukaryote Specific Primers
Source: PLoS One. 2014 Feb 7;9(2):e87624. doi: 10.1371/journal.pone.0087624 (PMC3917833; doi:10.1371/journal.pone.0087624)
Supplement: Table S3 — Start and end positions in the 18S rRNA gene, which was used to define the variable regions in Figure 2 . (DOCX) [file pone.0087624.s005.docx]

Table S3. Start and end positions in the 18S rRNA gene, which was used to define the variable regions in Figure 2.

| Region | V1 | V2 | V3 | V4 | V5 | V7 | V8 | V9 |
| --- | --- | --- | --- | --- | --- | --- | --- | --- |
| Start position | 40 | 167 | 395 | 575 | 971 | 1267 | 1460 | 1648 |
| End position | 167 | 395 | 575 | 971 | 1150 | 1460 | 1648 | 1788 |
